# Use of clinical scores in young Australian adults for prediction of atherosclerosis in middle age

**DOI:** 10.1186/s12872-023-03060-x

**Published:** 2023-02-03

**Authors:** Quan Huynh, Alison J. Venn, Costan G. Magnussen, Hong Yang, Prasanna Venkataraman, Terence Dwyer, Thomas H. Marwick

**Affiliations:** 1 Baker Heart and Diabetes Research Institute, 75 Commercial Road, Melbourne, VIC 3004 Australia; 2 Menzies Institute for Medical Research, University of Tasmania, Hobart, Australia; 3 Research Centre of Applied and Preventive Cardiovascular Medicine, Turku, Finland; 4 Centre for Population Health Research, Turku University Hospital, University of Turku, Turku, Finland; 5 Nuffield Department of Women’s & Reproductive Health, University of Oxford, Oxford, UK

**Keywords:** Atherosclerotic risk, Carotid plaque, Fuster BEWAT score, Ideal cardiovascular health score, Pooled cohort equation

## Abstract

We sought to apply a simple cardiovascular health tool not requiring laboratory tests (the Fuster-BEWAT score, FBS) to predict subclinical atherosclerosis. This study included 2657 young adults (< 40 years of age). In the prognostic group (n = 894, followed for 13 years until aged 40–50 years at follow-up), the primary outcome was presence of carotid plaque measured by carotid ultrasound at follow-up. Of these 894 participants, 86 (9.6%) had unilateral, and 23 participants (2.6%) had bilateral, carotid plaques at follow-up. The baseline FBS was predictive of carotid plaque at follow-up [odds ratio OR = 0.86 (95% CI 0.77–0.96) per 1-SD increase in FBS], similar to prediction from Pooled Cohort Equation [PCE, OR = 0.72 (0.61–0.85) per 1-SD decrease in PCE]. Risk scores at baseline predicted outcomes more strongly than those at follow-up, and did so independently of any changes over 13 years of follow-up. Similar discrimination for predicting carotid plaque after 13 years was found for both baseline FBS [C-statistic = 0.68 (95% CI 0.62–0.74)] and PCE [C-statistic = 0.69 (95% CI 0.63–0.75)]. Application of this FBS prognostic information to a contemporary cohort of 1763 young adults anticipates the future development of plaque in 305 (17.3%), especially in the 1494 participants (85%) with ≤ 2 metrics of ideal health. In conclusions, FBS measured in young adulthood predicted atherosclerosis 13 years later in middle age, independent of score changes over the follow-up period, emphasizing the importance of early damage to vascular health. FBS may be a simple and feasible risk score for engaging low-risk young people with reduction of future cardiovascular risk.

**Supplementary Information:**

The online version contains supplementary material available at 10.1186/s12872-023-03060-x.

## Introduction

A paradoxical age-based difference in the temporal evolution of atherosclerotic cardiovascular disease (ASCVD) mortality rates has emerged in recent years. For women and men aged ≥ 55 years, the annual coronary heart disease (CHD) mortality has continued a downward trend in age-adjusted mortality rates that have fallen to about one-third of their peak in the 1960s [1]. In contrast, women between the ages of 35–54 years showed a 1.5% increment in estimated annual mortality from 2000 to 2002 (compared to a 5.4% decrement from 1980 until 1989), and men in the same age group showed a 0.5% decrement from 2000 to 2002 (compared with a 6.2% decrement from 1980 to 1989) [2]. This observation has been repeated in different datasets and jurisdictions [3, 4], and has become part of the renewed focus on reducing the annual CHD burden of 775,000 hospitalizations and 75,000 deaths in the 35–64 years old age group in the USA [5].

A reduction of atherosclerotic burden in this age group would require an intervention in the preceding decades of life. Standard prediction tools for 10-years cardiovascular risk, such as the Pooled Cohort Equation (PCE, including age, sex, total cholesterol, high-density lipoprotein cholesterol, systolic blood pressure, smoking, and diabetes) [6], have been widely validated in different populations [7–9], but their focus is on the prediction of clinical events and risk factor management [10, 11] in adults aged 40–79 years and their utility in predicting subclinical atherosclerosis among young people is unclear. The Ideal Cardiovascular Health Score (ICHS) has been shown to be strongly and inversely associated with cardiovascular mortality and morbidity [7, 8], and is targeted at reducing cardiovascular risk in the general population [12]. However, in addition to its use of four modifiable life-style factors (smoking, body weight, physical activity and diet) and blood pressure, it also includes measurement of two traditional cardiovascular risk factors (total cholesterol, blood glucose) that require laboratory tests. The need for laboratory tests to derive the PCE and ICHS scores represents a barrier, because many people in their third decade do not engage with preventive and primary care services [13] or might not be offered such tests routinely. The Fuster-BEWAT Score (FBS) [14] is more attractive for use in young and low-risk individuals—first, because it focuses more on modifiable and behavioural factors (blood pressure, exercise, weight, alimentation and tobacco) and requires no laboratory tests, and second, because it integrates with lifestyle-based prevention of ASCVD.

In this study, we included data from 2657 adults from two population-based studies, 894 of whom were followed for 13 years. This study sought to estimate the risk profile among a contemporary cohort of young adults, to inform the risk of future ASCVD, based on the FBS.

## Methods

### Study design

This study included 2657 young Australian adults from two population-based studies who reported to be free of ASCVD. The first (prognostic) group were part of a nationally-representative sample derived from the Childhood Determinants of Adult Health (CDAH) study [15], which prospectively followed 894 participants aged 26–36 years in 2004–2006 to 2018–19 when they were aged 40–50 years. Participants who reported a history of angina, heart attack or stroke at baseline (n = 8) were excluded. Details of sampling procedures have been previously reported [15, 16]. The primary outcome was the presence of any carotid plaque, measured by carotid ultrasound at follow-up in 2018–2019. Secondary outcomes included the number of carotid arteries affected (none, unilateral or bilateral), maximum plaque thickness and maximum plaque area. All participants provided informed written consent. Ethics approval has been provided by University of Tasmania Human Research Ethics Committee. All data have been de-identified before analysis to protect participant privacy.

The second (application) group was derived from the REECE Study, a cross-sectional, population-based survey of employees and customers of a multi-national trade and building supplies company based in Australia. Participants completed an online CV risk assessment tool (the Ticker Test) and received guideline-recommended health prevention advice. Of the 4420 participants who completed the Ticker Test from January to April 2021, data was drawn from 1763 participants aged 18 to 40 years.

### Ultrasound measurements

Carotid measurements in all participants were made using semi-automated edge detection software (M’ath, Image Arena, Tomtec Imaging Systems GmbH), following standard guidelines [17]. Left and right common carotid artery, carotid bulb area and internal carotid artery were all examined to identify any carotid plaque. Maximum plaque diameter and area from all the examined segments of the carotid artery were recorded. All images from 2D vascular ultrasonography were analysed offline by a single observer who was blinded to the participant’s clinical data and characteristics. Plaques were defined as any focal structure encroaching into the arterial lumen by at least 0.5 mm or 50% of the surrounding intima-media thickness value (type I plaque), or demonstrating a thickness > 1.5 mm as measured from the intima-lumen interface to the media-adventitia interface (type II plaque) [17].

### Clinical assessment

This was gathered in all participants at the time of imaging, and also at follow-up in the prognostic group. Clinical data was obtained using questionnaires. Angina, heart attack and stroke were self-reported. Physical activity in the previous week was self-reported using the International Physical Activity Questionnaire [18]. Minutes/week spent on work-related, domestic and leisure-time physical activity at moderate and vigorous intensity was recorded together with time spent in active transport (classified as moderate intensity). Participants’ diets were recorded using a 127-item food frequency questionnaire [19]. Data on education and smoking were obtained through self-report at baseline and follow-up by using a questionnaire [15]. Education was classified into four categories: university or higher university degree, diploma certificate or equivalent, year 12 or equivalent and less than year 12.

Height and weight were measured at both time-points without headwear and heavy clothing. Body mass index was calculated as weight(kg)/[height(m)]^2^. Blood pressure was measured while sitting after at least a 5-min rest using an Omron HEM907 Blood Pressure Monitor (Omron Corporation, Kyoto, Japan) at baseline and follow-up. Biochemical parameters were measured in fasting blood samples by standard laboratory procedures [15].

### Cardiovascular health metrics

The FBS has five components, blood pressure (B), exercise (E), weight (W), alimentation (A) and tobacco (T), each of which was classified into four categories (from 0 to 3) as previously published [14]. All the components used to calculate the FBS are shown in Additional file 1: Table S1. Each component was then dichotomised as ideal (having maximal point of 3) or non-ideal (0–2 points). Therefore, the FBS ranged between 0 and 15 points, with more points implicating better health. In the process of calculating the FBS score for our participants, we have slightly modified the smoking component (“T”) to fit with our available data. Instead of classifying participants as “0” (> 1 pack of tobacco/day), “1” (< 1 pack of tobacco/day) and “3” (Non-smoker) as in the original publication, we classified our participants as “0” (Current smoker), “1” (Ex-smoker), and “3” (Never smoker). In the REECE study, questions regarding exercise, weight, alimentation and tobacco were consistent with FBS categories. However, systolic and diastolic BP was not collected; rather participants were asked if there was a prior diagnosis of hypertension and if it was controlled with anti-hypertensive therapy. Therefore, REECE participants who reported hypertension or taking anti-hypertensive medications were appointed “0” point for blood pressure, others were assumed to have an average blood pressure for their age group. The PCE score was calculated using a previously published equation that included seven components (age, sex, total cholesterol, high-density lipoprotein cholesterol, systolic blood pressure, smoking, and diabetes) [6]. These scores reflected 10-years risks of ASCVD and were presented as percentages of risk. Because higher PCE score implicates higher risks of ASCVD as opposed to that of FBS where higher scores implicate lower risks, a reverse of PCE score was used in analysis to facilitate easy comparison between the risk scores.

### Statistical analysis

Paired t-test was used to compare the components of risk scores between baseline and follow-up. Pearson correlation was used to estimate the correlation among the risk scores measured at both baseline and follow-up. Logistic regression was used to estimate the associations of the risk scores (FBS and PCE) and their individual components with the primary outcome (presence of carotid plaque at follow-up). Non-linear relationships between the primary outcome and predictors were tested and none was found. For the ease of comparing effect sizes, estimated risk scores were standardised to age and sex and the reported odds ratios were estimated per standard deviation in scores. The association of FBS with presence of carotid plaque in the prognostic group was applied to the application group to predict future carotid plaque in this sample. Changes in risk scores and their individual components were calculated as the differences between their values at baseline and those at follow-up. Participants were also classified as no carotid plaque, unilateral plaque (one side of carotid arteries) or bilateral plaques (both sides of carotid arteries). Maximal plaque thickness was classified into five categories: 0 (participants without any carotid plaque), 0.1–1.5 mm, 1.51–2.0 mm, 2.1–2.5 mm, and > 2.5 mm. Maximal plaque area was classified into five categories: 0 (participants without any carotid plaque), 0.1–5 mm^2^, 5.1–10 mm^2^, 10.1–20 mm^2^, and > 20 mm^2^. Ordinal logistic regression was used to estimate the associations of the risk scores and their individual components with these secondary outcomes. Odds ratios derived from an ordinal logistic regression is interpreted the same as in a binary model, which is the change in odds per unit increase in risk scores. Comparisons between the areas under the receiver operating characteristic curves were performed using the Hanley and McNeil method [20]. Calibration of each risk score in predicting the study primary outcome was performed by plotting observed and predicted risks per decile of estimated scores. STATA 17 (StataCorp, College Station, TX) was used for data analysis. A *p* < 0.05 was used to define statistical significance in this study.

## Results

*Participant characteristics* at both baseline and follow-up are shown in Table 1. One in four participants from the application study (REECE) was obese, which was a much greater proportion (*p* < 0.001) than that in the original prognostic study (CDAH) at a similar age range 15 years earlier in 2004–2006. Although the application study participants were less likely to smoke, they consumed less fruit and vegetables and were more likely to be hypertensive than their counterparts in the prognostic study. This was translated into higher risk profiles in the application study participants, with a significantly lower average FBS score compared with that in the CDAH participants (8.5 vs 11.4, *p* < 0.001).

**Table 1:** Participant characteristics at baseline and follow-up

	CDAH (n = 894)		REECE (n = 1763)
	Baseline (2004–2006)	Follow-up (2018–19)	Baseline (2021)
*Sociodemographic*			
Age range (year)	26–36	40–50	18–40
Male	48.0%	48.0%	85.9%
*Body size*			
BMI (kg/m^2^)	25.3 ± 4.5	27.2 ± 5.3	27.7 ± 5.3
*Obesity status*			
Normal weight	54.0%	37.7%	31.2%
Over weight	34.3%	39.9%	44.0%
Obese	11.7%	22.4%	24.8%
*Self-reported leisure physical activity*			
Moderate (min/week)	0 [0, 25]	0 [0, 31]	45 [0, 105]
Vigorous (min/week)	0 [0, 100]	0 [0, 120]	15 [0, 45]
*Smoking*			
Current smoker	23%	9%	15%
Ex-smoker	21%	21%	20%
Non-smoker	56%	70%	65%
*Diet*			
Fruit and vegetable serves/day	3.6 ± 1.9	3.7 ± 1.9	2.4 ± 1.5
*Blood tests*			
TC (mmol/L)	4.9 ± 0.9	5.2 ± 0.9	n/a
HDL-C (mmol/L)	1.4 ± 0.3	1.5 ± 0.4	n/a
LDL-C (mmol/L)	3.0 ± 0.8	3.1 ± 0.8	n/a
Triglycerides (mmol/L)	1.1 ± 0.6	1.2 ± 0.8	n/a
Fasting glucose (mmol/L)	5.0 ± 0.5	4.8 ± 0.8	n/a
*Medications*			
Any cholesterol lowering medication	0.4%	1.6%	0.2%
Any blood pressure lowering medication	0.5%	5%	1.1%
*FBS*			
Total score	11.4 ± 2.4	10.9 ± 2.7	8.5 ± 2.3
*Number of ideal metrics in FBS*			
0	0.7%	0.8%	7.0%
1	7.0%	5.8%	45.0%
2	22.3%	20.5%	32.8%
3	34.4%	34.8%	13.1%
4	26.5%	27.7%	2.2%
5	9.1%	10.4%	0%

Data are shown either as either mean ± standard deviation or median [quartiles] for continuous variables and as % (n) for categorical variables

*BMI* body mass index, *SBP* systolic blood pressure, *DBP* diastolic blood pressure, *PWC* physical work capacity, *TC* total cholesterol, *HDL-C* high density lipoprotein cholesterol, *LDL-C* low density lipoprotein cholesterol, *FBS* Fuster-BEWAT Score

Our subsequent step investigated whether FBS measured at baseline during young adulthood could predict future ASCVD in the CDAH participants who were followed-up for 13 years, and how many of the REECE participants are predicted to have ASCVD in the future based on their risk profiles estimated by FBS.

### Study outcomes

At follow-up, unilateral carotid plaque was present in 88 CDAH participants (10.4%) and bilateral carotid plaque in 23 participants (2.6%). Maximal plaque thickness was 2.0(0.6) mm, and maximal carotid plaque area was 15.4(11.5) mm^2^. A detailed description of these carotid plaques is shown in Additional file 1: Table S2.

### Fuster-BEWAT score (FBS)

Of all the FBS components at baseline in CDAH, the most common in the ideal category was blood pressure, followed by exercise. At follow-up, non-smoking had the highest prevalence in the ideal category, also followed by exercise. There were significant differences between baseline and follow-up regarding blood pressure, body weight, alimentation and smoking (all *p* < 0.05), but not exercise (*p* = 0.17). Specifically, participants were more likely to be hypertensive and overweight or obese, consume more fruit and vegetables and were less likely to smoke at follow-up than at baseline. FBS measured at baseline and follow-up were moderately correlated (r = 0.46, *p* < 0.001).

**Fig. 1:**
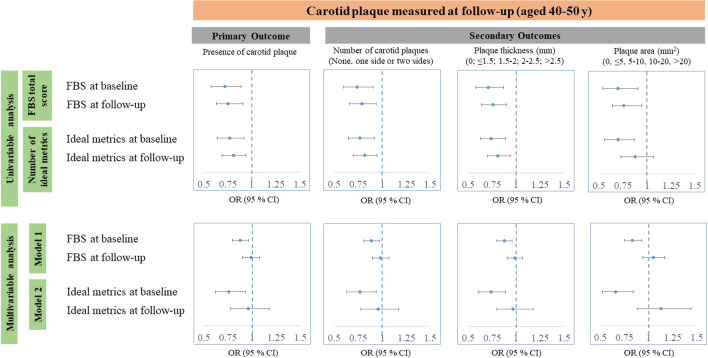
Associations of Fuster-BEWAT score at baseline and follow-up with carotid plaque at follow-up

Figure 1 shows the associations of FBS at baseline and follow-up with the primary and secondary study outcomes. Both higher total score of FBS and number of ideal metrics were associated with lower odds of having a carotid plaque in 13 years. The effect sizes of FBS measured at baseline were greater than those of FBS measured at follow-up. In mutual adjustment, only FBS measured at baseline remained as a significant predictor of carotid plaque at follow-up, implicating the importance of risk measured early in life over that of current risk or changes in risk during the follow-up.

All these analyses in Fig. 1 were repeated for the individual components of FBS and findings are shown in Additional file 1: Tables S3 and S4. Similar to the findings from Fig. 1, most of these individual components at baseline had greater effect sizes in predicting primary and secondary outcomes than those at follow-up, and were associated with study outcomes independent of changes over 13 years. The exceptions were diet and smoking, where measures at follow-up were more strongly associated with outcomes than those at baseline, and physical activity where neither baseline nor follow-up measures were associated with outcomes.

### FBS and other health metrics

**Fig. 2:**
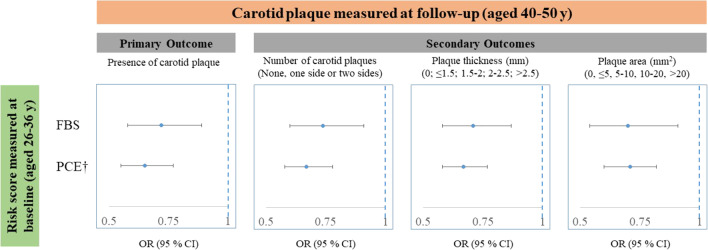
Univariable associations of three risk scores at baseline with carotid plaque at follow-up, using data from the CDAH study. Abbreviations: FBS (Fuster-BEWAT), PCE (Pooled Cohort Equations). †The effect size reflects one standard deviation increase in FBS and one standard deviation decrease in PCE

**Fig. 3:**
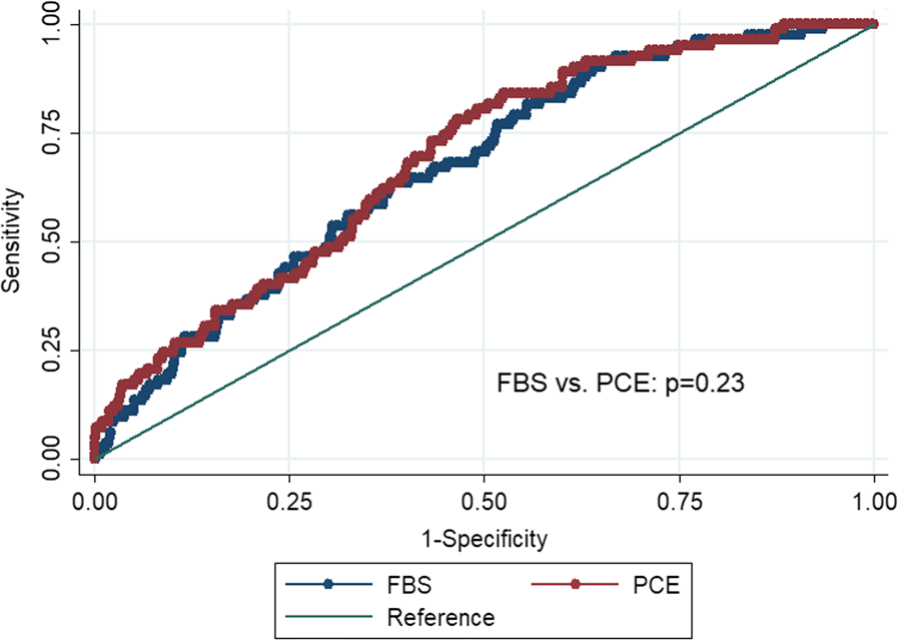
Areas under the curve of the three risk scores in predicting future carotid plaque, using data from the CDAH study. Abbreviations: FBS (Fuster-BEWAT), PCE (Pooled Cohort Equations)

The FBS showed a negative correlation with PCE (r = − 0.36). Figure 2 shows modest differences in the effect sizes of the FBS and PCE at baseline in predicting carotid plaque at follow-up. Table 2 and Fig. 3 shows similar discrimination and incremental values of the scores in predicting the presence of a future carotid plaque to that of the base model which included age and sex. Excluding participants who took cholesterol lowering medications at both baseline and follow-up showed the same associations of both FBS and PCE with carotid plaque. Additional file 1: Fig. S2 reports the calibration of the risk scores at baseline in predicting carotid plaque at follow-up. In general, the scores produced a slight overestimation of risks (with FBS least so), with very strong correlations between predicted and observed risks (R-squared = 0.87 for FBS and 0.79 for PCE). Additional file 1: Table S5 shows no improvement in reclassification of risks when adding PCE to FBS in predicting carotid plaque (net reclassification index − 0.0062, *p* = 0.88).

**Table 2:** Predicting presence of carotid plaque 13 years later in mid-adulthood

	Model 0: base model AUC = 0.62 (0.55, 0.67)	Model 1: FBS AUC = 0.68 (0.62, 0.74)	Model 2: PCE AUC = 0.69 (0.63, 0.75)
	OR (95% CI)	OR (95% CI)	OR (95% CI)
Age	1.08 (1.00, 1.17)	1.09 (1.00, 1.18)	1.07 (0.99, 1.16)
Male sex	2.01 (1.33, 3.02)	1.82 (1.08, 3.06)	1.64 (1.06, 2.52)
FBS when aged 26–36 years^†^		0.86 (0.77, 0.96)	
PCE when aged 26–36 years^†^			0.72 (0.61, 0.85)
	Reference	Model 1 versus model 0: *p* = 0.028	Model 2 versus model 0: *p* = 0.011

*FBS* Fuster-BEWAT Score, *PCE* Pooled Cohort Equations, *AUC* area under the curve, *OR* odds ratio

^†^The effect size reflects one standard deviation increase in FBS and one standard deviation decrease in PCE

### Implications of FBS in the current group

Table 3 summarises the distribution of participants according to FBS score in each group, the observed frequency of carotid plaque in the prognostic group, and the predicted frequency in the application group. The development of subclinical atherosclerosis can be anticipated in 18% of participants with ≤ 2 ideal metrics, compared with < 10% of those with 4–5 ideal metrics.

**Table 3:** Prediction of future carotid plaque in the REECE study using FBS

	Number of ideal metrics in FBS			
	0–1	2	3	4–5
Observed carotid plaque in prognostic group (CDAH)	18.3% (11/60)	18.4% (34/185)	11.6% (34/292)	9.5% (30/316)
Predicted carotid plaque in application group	168/916	106/578	27/231	4/38

## Discussion

This study investigated the utility of the FBS among young, low-risk adults in predicting the presence and extent of carotid plaques in middle age and has several important findings. First, FBS applied in young adulthood was predictive of the presence and extent of carotid plaques 13 years later in mid-adulthood, highlighting the importance of early-life risk factors to the presence and extent of atherosclerosis. Second, risk factors in young adulthood predict the presence of future carotid plaques, independent of current risk factors or changes in these risk factors during follow-up. Third, FBS appeared to have similar discrimination in predicting future carotid plaques in young and low-risk adults as that of PCE that require laboratory tests. Finally, subclinical atherosclerosis can be anticipated in 18% of participants with ≤ 2 ideal metrics, compared with < 10% of those with 4–5 ideal metrics.

### Predictors of carotid plaque

Among the individual components of the risk scores, consistent predictors of the presence and extent of carotid plaques included blood pressure, body mass index, blood cholesterol, blood glucose, diet and smoking. Age and male sex were also predictive of the presence of a carotid plaque. While these factors are well-known predictors of ASCVD, body mass index is not included in some risk scores, such as the PCE. This reflects previous findings that although body mass index was associated with cardiovascular and all-cause mortality [21, 22], it did not add an incremental value to blood pressure, diabetes and cholesterol in the prediction of cardiovascular disease [23]. Baseline measures of blood pressure, body mass index, blood cholesterol and blood glucose were more strongly associated with outcomes than those at follow-up, likely reflecting long-lasting damage of these risk factors to cardiovascular health. In contrast, measures of diet and smoking at follow-up were more predictive of outcomes than those at baseline, likely reflecting the more modifiable nature of these risk factors. Of all the risk factors, diet and smoking had the greatest improvements over the 13 years of follow-up. Physical activity was not associated with carotid plaques in our study, but was reported to be associated with all-cause and cardiovascular mortality in previous studies [24]. This discrepancy between our and previous findings can be explained by differences in the outcomes of interest and the populations being studied. While our study focused on predicting the early phase of ASCVD in young adults, previous studies investigated advanced ASCVD and mortality in middle-aged to older adults.

It is an important finding that risk scores measured in young adulthood predicted the presence of carotid plaques 13 years later, but that changes in these scores and their individual components added little to the prediction of atherosclerosis. These findings could be partly explained by the modest changes in these risk factors over the 13-years follow-up period, which also appears to be common in other countries such as the United States [25]. However, the effect sizes for the associations of FBS and most of its individual components at baseline were consistently greater than those at follow-up, which suggests that damage to the vascular system may have been determined by lifestyle behaviours and risk factors early in life. These speculations are further supported by our findings that levels of blood cholesterol in young adulthood were among the strongest predictors of atherosclerosis and that they could predict the presence and extent of carotid plaques later in life independent of any of their changes over the follow-up period. Regardless of a small increase in cholesterol lowering medication use from baseline to follow-up that implicated disease progression, FBS at baseline was independently associated with carotid plaque at follow-up. Our findings are consistent with those from the Coronary Artery Risk Development in Young Adults cohort study, that risk scores measured in generally healthy adults aged 18–30 years could predict 25-years risks of fatal and non-fatal events due to ASCVD [26]. Also in relation to this context, previous findings from our group and others have provided consistent evidence on the independent long-term effects of obesity, metabolic syndrome, blood pressure, blood cholesterol and exposure to parental smoking in childhood on vascular health in adulthood. [15, 27–30].

### Comparison among different screening tools

Both screening tools included in this study (FBS and PCE) performed relatively well and had similar discriminatory power when used to predict future carotid plaques among young and low-risk adults. Although these tools share several components (blood pressure and smoking), the PCE also requires laboratory tests to measure blood cholesterol and blood glucose. For this reason, the FBS may be considered a more feasible option to foster primary prevention in youth or low-risk individuals where laboratory tests are not feasible or available. While routine screening for cardiovascular risk factors by laboratory tests is still recommended where possible, the FBS is particularly useful for screening for risks of ASCVD in non-clinical environments, for self-screening and in remote areas or low-income countries where laboratory testing is less accessible. Our findings are further supported by previous findings from large prospective cohorts such as the National Health and Nutrition Examination Survey and the International Childhood Cardiovascular Cohorts Consortium that non-laboratory-based risk factors may predict clinical and preclinical cardiovascular disease as accurately as laboratory risk factors [29, 31]. All the components of the FBS could be easily obtained within five minutes. Although physical activity and diet are usually measured by sophisticated and validated questionnaires for research purpose, the FBS only requires simple measurements that could be obtained using a single questionnaire. The other components of the FBS (blood pressure, body weight and smoking) are usually measured routinely at most clinics, which makes the FBS highly feasible for clinical use.

### Strengths and limitations

This study was a 13-years follow-up of a large population-based sample of young adults with measurement of an extensive range of study factors. Loss to follow-up has occurred, with non-participants at follow-up having greater body mass index and lower education at baseline. Nonetheless, this investigation represents a large sample from a well-characterised study population for which the distributional range of study factors, confounders and effect modifiers was not restricted by sampling or diminished by attrition. Threats to external validity are less of an issue in these circumstances [32]. Another strength of this study was the use of contemporary imaging technology and a single reader for all ultrasound images in our study. Using carotid plaque as the primary endpoint enabled investigation of subclinical cardiovascular disease in a relatively young and low-risk population in whom early preventive intervention might lead to greater benefits.

A limitation of this study was the lack of plaque measurement at baseline, which has limited our ability to quantify the development or progress of atherosclerosis throughout the follow-up period. However, our participants were young and likely had a low prevalence of carotid plaques at baseline. This speculation was supported by very low baseline values of carotid intima-media thickness as reported previously [15]. We did not assess plaque morphology in our study and therefore could not comment on the associations of FBS or any of its components with plaque characteristics. Many lifestyle factors such as physical activity and diet are difficult to quantify. Although our measurements of these factors were consistent with those used in the risk scores that we investigated in this study, they were not the current best measurements available for physical activity and diet. This limitation might have underestimated the associations of these factors with the study outcomes. While blood pressure was measured consistently at both baseline and follow-up in the derivation cohort, it was estimated based on participant medical history of hypertension and anti-hypertensive medication use. This might have underestimated the association of FBS with carotid plaque reported in this study for the validation cohort. In addition, although we investigated subclinical atherosclerosis (which precedes clinical atherosclerosis), further research is required to determine whether low-risk individuals will have long-term benefits from intensive intervention at an early stage to prevent subclinical atherosclerosis.

### Conclusions

FBS measured in young adulthood predicted subclinical atherosclerosis 13 years later independently of any changes over the follow-up period, suggesting vascular damage occurred early in life. The discrimination of FBS in predicting the presence and extent of future carotid plaques in middle age was similar to other risk scores that require laboratory tests. These findings highlight the benefits of using FBS as a simpler and more feasible risk score for predicting future risks of ASCVD in young individuals.

## Supplementary Information

**Additional file 1. Supplementary Tables and Figures. Supplementary Table 1.** Estimation of Fuster-BEWAT score. **Supplementary Table 2.** Characteristics of carotid plaque measured at follow-up when participants were aged 40-50 years. **Supplementary Table 3.** Univariable logistic regression of risk score components with carotid plaque, with adjustment for changes over 13 years of follow-up. **Supplementary Table 4.** Logistic regression of risk score components with carotid plaque, with adjustment for changes over 13 years of follow-up. **Supplementary Table 5.** Net Reclassification of adding PCE to FBS in prediction of carotid plaque at follow-up. **Supplementary Figure 1.** Study sample. **Supplementary Figure 2.** Calibration of the 3 risk scores in predicting carotid plaque at follow-up.

## Data Availability

The datasets used and/or analysed during the current study available from the corresponding author on reasonable request.

## References

[1] Mensah GA, Wei GS, Sorlie PD, Fine LJ, Rosenberg Y, Kaufmann PG, Mussolino ME, Hsu LL, Addou E, Engelgau MM, Gordon D (2017). Decline in cardiovascular mortality: possible causes and implications. Circ Res.

[2] Ford ES, Capewell S (2007). Coronary heart disease mortality among young adults in the U.S. from 1980 through 2002: concealed leveling of mortality rates. J Am Coll Cardiol.

[3] O'Flaherty M, Allender S, Taylor R, Stevenson C, Peeters A, Capewell S (2012). The decline in coronary heart disease mortality is slowing in young adults (Australia 1976–2006): a time trend analysis. Int J Cardiol.

[4] Wilmot KA, O'Flaherty M, Capewell S, Ford ES, Vaccarino V (2015). Coronary heart disease mortality declines in the United States from 1979 through 2011: evidence for stagnation in young adults, especially women. Circulation.

[5] Prevention CfDC a. Heart disease and stroke deaths hitting middle age adults in large numbers. 2018; 2020.

[6] Goff DC, Lloyd-Jones DM, Bennett G, Coady S, D'Agostino RB, Gibbons R, Greenland P, Lackland DT, Levy D, O'Donnell CJ, Robinson JG, Schwartz JS, Shero ST, Smith SC, Sorlie P, Stone NJ, Wilson PW, Jordan HS, Nevo L, Wnek J, Anderson JL, Halperin JL, Albert NM, Bozkurt B, Brindis RG, Curtis LH, DeMets D, Hochman JS, Kovacs RJ, Ohman EM, Pressler SJ, Sellke FW, Shen WK, Smith SC, Tomaselli GF (2014). 2013 ACC/AHA guideline on the assessment of cardiovascular risk: a report of the American College of Cardiology/American Heart Association Task Force on Practice Guidelines. Circulation.

[7] Guo L, Zhang S (2017). Association between ideal cardiovascular health metrics and risk of cardiovascular events or mortality: a meta-analysis of prospective studies. Clin Cardiol.

[8] Fang N, Jiang M, Fan Y (2016). Ideal cardiovascular health metrics and risk of cardiovascular disease or mortality: a meta-analysis. Int J Cardiol.

[9] Muntner P, Colantonio LD, Cushman M, Goff DC, Howard G, Howard VJ, Kissela B, Levitan EB, Lloyd-Jones DM, Safford MM (2014). Validation of the atherosclerotic cardiovascular disease Pooled Cohort risk equations. JAMA.

[10] Whelton PK, Carey RM, Aronow WS, Casey DE, Collins KJ, Dennison Himmelfarb C, DePalma SM, Gidding S, Jamerson KA, Jones DW, MacLaughlin EJ, Muntner P, Ovbiagele B, Smith SC, Spencer CC, Stafford RS, Taler SJ, Thomas RJ, Williams KA, Williamson JD, Wright JT (2018). 2017 ACC/AHA/AAPA/ABC/ACPM/AGS/APhA/ASH/ASPC/NMA/PCNA Guideline for the Prevention, Detection, Evaluation, and Management of High Blood Pressure in Adults: A Report of the American College of Cardiology/American Heart Association Task Force on Clinical Practice Guidelines. J Am Coll Cardiol.

[11] Grundy SM, Stone NJ, Bailey AL, Beam C, Birtcher KK, Blumenthal RS, Braun LT, de Ferranti S, Faiella-Tommasino J, Forman DE, Goldberg R, Heidenreich PA, Hlatky MA, Jones DW, Lloyd-Jones D, Lopez-Pajares N, Ndumele CE, Orringer CE, Peralta CA, Saseen JJ, Smith SC, Sperling L, Virani SS, Yeboah J (2019). 2018 AHA/ACC/AACVPR/AAPA/ABC/ACPM/ADA/AGS/APhA/ASPC/NLA/PCNA guideline on the management of blood cholesterol: a report of the American College of Cardiology/American Heart Association Task Force on Clinical Practice Guidelines. Circulation.

[12] Lloyd-Jones DM, Hong Y, Labarthe D, Mozaffarian D, Appel LJ, Van Horn L, Greenlund K, Daniels S, Nichol G, Tomaselli GF, Arnett DK, Fonarow GC, Ho PM, Lauer MS, Masoudi FA, Robertson RM, Roger V, Schwamm LH, Sorlie P, Yancy CW, Rosamond WD (2010). Defining and setting national goals for cardiovascular health promotion and disease reduction: the American Heart Association's strategic Impact Goal through 2020 and beyond. Circulation.

[13] Australian Institute of Health and Welfare 2018. Medicare Benefits Schedule GP and specialist attendances and expenditure in 2016–17 (Cat. no: HPF 30).

[14] Gomez-Pardo E, Fernandez-Alvira JM, Vilanova M, Haro D, Martinez R, Carvajal I, Carral V, Rodriguez C, de Miguel M, Bodega P, Santos-Beneit G, Penalvo JL, Marina I, Perez-Farinos N, Dal Re M, Villar C, Robledo T, Vedanthan R, Bansilal S, Fuster V (2016). A comprehensive lifestyle peer group-based intervention on cardiovascular risk factors: the randomized controlled fifty-fifty program. J Am Coll Cardiol.

[15] Huynh Q, Blizzard L, Sharman J, Magnussen C, Schmidt M, Dwyer T, Venn A (2013). Relative contributions of adiposity in childhood and adulthood to vascular health of young adults. Atherosclerosis.

[16] Dwyer T, Gibbons LE (1994). The Australian Schools Health and Fitness Survey: physical fitness related to blood pressure but not lipoproteins. Circulation.

[17] Touboul PJ, Hennerici MG, Meairs S, Adams H, Amarenco P, Bornstein N, Csiba L, Desvarieux M, Ebrahim S, Hernandez Hernandez R, Jaff M, Kownator S, Naqvi T, Prati P, Rundek T, Sitzer M, Schminke U, Tardif JC, Taylor A, Vicaut E, Woo KS (2012). Mannheim carotid intima-media thickness and plaque consensus (2004-2006-2011): an update on behalf of the advisory board of the 3rd, 4th and 5th watching the risk symposia, at the 13th, 15th and 20th European Stroke Conferences, Mannheim, Germany, 2004, Brussels, Belgium, 2006, and Hamburg, Germany, 2011. Cerebrovasc Dis.

[18] Craig CL, Marshall AL, Sjostrom M, Bauman AE, Booth ML, Ainsworth BE, Pratt M, Ekelund U, Yngve A, Sallis JF, Oja P (2003). International physical activity questionnaire: 12-country reliability and validity. Med Sci Sports Exerc.

[19] Wilson JE, Blizzard L, Gall SL, Magnussen CG, Oddy WH, Dwyer T, Venn AJ, Smith KJ (2019). An age- and sex-specific dietary guidelines index is a valid measure of diet quality in an Australian cohort during youth and adulthood. Nutr Res.

[20] Hanley JA, McNeil BJ (1983). A method of comparing the areas under receiver operating characteristic curves derived from the same cases. Radiology.

[21] Di Angelantonio E, Bhupathiraju ShN, Wormser D, Gao P, Kaptoge S, Berrington de Gonzalez A, Cairns BJ, Huxley R, Jackson ChL, Joshy G, Lewington S, Manson JE, Murphy N, Patel AV, Samet JM, Woodward M, Zheng W, Zhou M, Bansal N, Barricarte A, Carter B, Cerhan JR, Smith GD, Fang X, Franco OH, Green J, Halsey J, Hildebrand JS, Jung KJ, Korda RJ, McLerran DF, Moore SC, O'Keeffe LM, Paige E, Ramond A, Reeves GK, Rolland B, Sacerdote C, Sattar N, Sofianopoulou E, Stevens J, Thun M, Ueshima H, Yang L, Yun YD, Willeit P, Banks E, Beral V, Chen Z, Gapstur SM, Gunter MJ, Hartge P, Jee SH, Lam TH, Peto R, Potter JD, Willett WC, Thompson SG, Danesh J, Hu FB, Global BMIMC (2016). Body-mass index and all-cause mortality: individual-participant-data meta-analysis of 239 prospective studies in four continents. Lancet.

[22] Khan SS, Ning H, Wilkins JT, Allen N, Carnethon M, Berry JD, Sweis RN, Lloyd-Jones DM (2018). Association of body mass index with lifetime risk of cardiovascular disease and compression of morbidity. JAMA Cardiol.

[23] Wormser D, Kaptoge S, Di Angelantonio E, Wood AM, Pennells L, Thompson A, Sarwar N, Kizer JR, Lawlor DA, Nordestgaard BG, Ridker P, Salomaa V, Stevens J, Woodward M, Sattar N, Collins R, Thompson SG, Whitlock G, Danesh J (2011). Separate and combined associations of body-mass index and abdominal adiposity with cardiovascular disease: collaborative analysis of 58 prospective studies. Lancet.

[24] Ekelund U, Tarp J, Steene-Johannessen J, Hansen BH, Jefferis B, Fagerland MW, Whincup P, Diaz KM, Hooker SP, Chernofsky A, Larson MG, Spartano N, Vasan RS, Dohrn IM, Hagströmer M, Edwardson C, Yates T, Shiroma E, Anderssen SA, Lee IM (2019). Dose-response associations between accelerometry measured physical activity and sedentary time and all cause mortality: systematic review and harmonised meta-analysis. BMJ.

[25] Huffman MD, Capewell S, Ning H, Shay CM, Ford ES, Lloyd-Jones DM (2012). Cardiovascular health behavior and health factor changes (1988–2008) and projections to 2020: results from the National Health and Nutrition Examination Surveys. Circulation.

[26] Gooding HC, Ning H, Gillman MW, Shay C, Allen N, Goff DC, Lloyd-Jones D, Chiuve S (2017). Application of a lifestyle-based tool to estimate premature cardiovascular disease events in young adults: the coronary artery risk development in young adults (CARDIA) study. JAMA Intern Med.

[27] Gall S, Huynh QL, Magnussen CG, Juonala M, Viikari JS, Kahonen M, Dwyer T, Raitakari OT, Venn A (2014). Exposure to parental smoking in childhood or adolescence is associated with increased carotid intima-media thickness in young adults: evidence from the Cardiovascular Risk in Young Finns study and the Childhood Determinants of Adult Health Study. Eur Heart J.

[28] Koskinen J, Juonala M, Dwyer T, Venn A, Petkeviciene J, Čeponienė I, Bazzano L, Chen W, Sabin MA, Burns TL, Viikari JSA, Woo JG, Urbina EM, Prineas R, Hutri-Kähönen N, Sinaiko A, Jacobs DR, Steinberger J, Daniels S, Raitakari O, Magnussen CG (2019). Utility of different blood pressure measurement components in childhood to predict adult carotid intima-media thickness. Hypertension.

[29] Koskinen J, Juonala M, Dwyer T, Venn A, Thomson R, Bazzano L, Berenson GS, Sabin MA, Burns TL, Viikari JSA, Woo JG, Urbina EM, Prineas R, Hutri-Kähönen N, Sinaiko A, Jacobs D, Steinberger J, Daniels S, Raitakari OT, Magnussen CG (2018). Impact of lipid measurements in youth in addition to conventional clinic-based risk factors on predicting preclinical atherosclerosis in adulthood: international childhood cardiovascular cohort consortium. Circulation.

[30] Koskinen J, Magnussen CG, Sinaiko A, Woo J, Urbina E, Jacobs DR, Steinberger J, Prineas R, Sabin MA, Burns T, Berenson G, Bazzano L, Venn A, Viikari JSA, Hutri-Kähönen N, Raitakari O, Dwyer T, Juonala M (2017). Childhood age and associations between childhood metabolic syndrome and adult risk for metabolic syndrome, type 2 diabetes mellitus and carotid intima media thickness: the international childhood cardiovascular cohort consortium. J Am Heart Assoc.

[31] Gaziano TA, Young CR, Fitzmaurice G, Atwood S, Gaziano JM (2008). Laboratory-based versus non-laboratory-based method for assessment of cardiovascular disease risk: the NHANES I follow-up study cohort. Lancet.

[32] Miettinen O (1985). Theoretical epidemiology. Principles of occurrence research in medicine.

