# Supplementary material for: Use of clinical scores in young Australian adults for prediction of atherosclerosis in middle age
Source: BMC Cardiovasc Disord. 2023 Feb 3;23:63. doi: 10.1186/s12872-023-03060-x (PMC9896707; doi:10.1186/s12872-023-03060-x)
Supplement: Supplementary file 1 — Additional file 1. Supplementary Tables and Figures. Supplementary Table 1. Estimation of Fuster-BEWAT score. Supplementary Table 2. Characteristics of carotid plaque measured at follow-up when participants were aged 40-50 years. Supplementary Table 3. Univariable logistic regression of risk score components with carotid plaque, with adjustment for changes over 13 years of follow-up. Supplementary Table 4. Logistic regression of risk score components with carotid plaque, with adjustment for changes over 13 years of follow-up. Supplementary Table 5. Net Reclassification of adding PCE to FBS in prediction of carotid plaque at follow-up. Supplementary Figure 1. Study sample. Supplementary Figure 2. Calibration of the 3 risk scores in predicting carotid plaque at follow-up. [file 12872_2023_3060_MOESM1_ESM.docx]

| **Supplementary Table 1.** Estimation of Fuster-BEWAT score | |
| --- | --- |
|  |  |
|  | Fuster-BEWAT score |
| Blood pressure |  |
| SBP≥140 and/or DBP≥90mmHg | 0 |
| SBP 130-139 and/or DBP 85-89mmHg | 1 |
| SBP 120-129 and/or DBP 80-84mmHg | 2 |
| SBP<120 and DBP<80mmHg) | 3 |
| Exercise |  |
| <10 min/week of MVPA | 0 |
| 10-75 min/week of MVPA | 1 |
| 75-149 min/week of MVPA | 2 |
| ≥150 min/week of MVPA | 3 |
| Weight |  |
| BMI ≥30kg/m^2^ | 0 |
| BMI 25-29.99kg/m^2^ | 1 |
| BMI <25kg/m^2^) | 3 |
| Alimentation |  |
| <1 fruit/vegetable serving daily | 0 |
| 1-2 fruit/vegetable servings daily | 1 |
| 3-4 fruit/vegetable servings daily | 2 |
| >4 fruit/vegetable servings daily | 3 |
| Tobacco |  |
| Current smoker | 0 |
| Ex-smoker | 1 |
| Non-smoker | 3 |
| Abbreviations: SBP (systolic blood pressure), DBP (diastolic blood pressure), MVPA (moderate or vigorous physical activity), BMI (body mass index). | |

| **Supplementary Table 2.** Characteristics of carotid plaque measured at follow-up when participants were aged 40-50 years | | | |
| --- | --- | --- | --- |
|  |  | Left carotid plaque | Right carotid plaque |
| Presence of plaque | | n=71 | n=61 |
| Maximum thickness (mm) | | 2.0±0.6 | 2.0±0.5 |
| Maximum area (mm^2^) | | 10.7±9.8 | 12.1±9.6 |
| Location of plaque | Bulb | n=62 | n=51 |
|  | ICA | n=8 | n=7 |
|  | Both bulb and ICA or CCA | n=1 | n=3 |
| Type of plaque | I | n=44 | n=45 |
|  | II | n=27 | n=16 |
| Abbreviations: ICA (internal carotid artery), CCA (common carotid artery) | | | |

| **Supplementary Table 3**. Univariable logistic regression of risk score components with carotid plaque, with adjustment for changes over 13 years of follow-up | | | | | |
| --- | --- | --- | --- | --- | --- |
|  | | Carotid plaque when aged 40-50 years | | | |
|  | | Presence of carotid plaque | Number of carotid plaque  (None, one side or two sides) | Plaque thickness (mm)  (0; ≤1.5; 1.5-2; 2-2.5; >2.5) | Plaque area (mm^2^)  (0, ≤5, 5-10, 10-20, >20) |
| **Individual components of risk scores** | | OR (95% CI) | OR (95% CI) | OR (95% CI) | OR (95% CI) |
| SBP | When aged 26-36 years | **1.03 (1.02, 1.05)** | **1.03 (1.02, 1.05)** | **1.03 (1.01, 1.04)** | **1.03 (1.01, 1.05)** |
|  | When aged 40-50 years | **1.02 (1.01, 1.03)** | **1.02 (1.01, 1.03)** | **1.02 (1.01, 1.03)** | 1.01 (0.99, 1.02) |
| DBP | When aged 26-36 years | **1.03 (1.01, 1.05)** | **1.03 (1.01, 1.05)** | **1.03 (1.01, 1.05)** | **1.03 (1.01, 1.06)** |
|  | When aged 40-50 years | **1.02 (1.01, 1.04)** | **1.02 (1.01, 1.04)** | **1.02 (1.01, 1.04)** | **1.02 (1.00, 1.04)** |
| BMI | When aged 26-36 years | **1.06 (1.01, 1.10)** | **1.05 (1.01, 1.09)** | **1.05 (1.02, 1.10)** | 1.03 (0.98, 1.08) |
|  | When aged 40-50 years | 1.03 (1.00, 1.06) | 1.02 (0.99, 1.05) | 1.03 (0.99, 1.06) | 1.00 (0.97, 1.04) |
| Physical activity | When aged 26-36 years | 1.00 (1.00, 1.00) | 1.00 (1.00, 1.00) | 1.00 (0.99, 1.00) | 1.00 (1.00, 1.00) |
|  | When aged 40-50 years | 1.00 (1.00, 1.00) | 1.00 (1.00, 1.00) | 1.00 (1.00, 1.00) | 1.00 (1.00, 1.00) |
| Diet* | When aged 26-36 years | 0.99 (0.89, 1.09) | 0.98 (0.89, 1.08) | 0.99 (0.90, 1.08) | 0.95 (0.84, 1.07) |
|  | When aged 40-50 years | **0.91 (0.82, 0.99)** | **0.91 (0.83, 0.99)** | 0.92 (0.84, 1.00) | **0.88 (0.79, 0.99)** |
| Smoking† | When aged 26-36 years | **0.81 (0.68, 0.97)** | **0.83 (0.70, 0.98)** | **0.80 (0.68, 0.95)** | **0.79 (0.64, 0.97)** |
|  | When aged 40-50 years | **0.79 (0.65, 0.96)** | **0.79 (0.65, 0.96)** | **0.78 (0.65, 0.95)** | **0.74 (0.59, 0.92)** |
| TC | When aged 26-36 years | **1.40 (1.16, 1.69)** | **1.37 (1.15, 1.65)** | **1.42 (1.19, 1.70)** | **1.44 (1.18, 1.68)** |
|  | When aged 40-50 years | **1.36 (1.12, 1.56)** | **1.34 (1.13, 1.57)** | **1.33 (1.18, 1.52)** | **1.31 (1.10, 1.51)** |
| HDL | When aged 26-36 years | **0.39 (0.20, 0.78)** | **0.42 (0.22, 0.80)** | **0.47 (0.26, 0.88)** | **0.28 (0.12, 0.66)** |
|  | When aged 40-50 years | **0.49 (0.30, 0.79)** | **0.51 (0.32, 0.80)** | **0.53 (0.34, 0.83)** | **0.53 (0.30, 0.95)** |
| LDL | When aged 26-36 years | **1.69 (1.36, 2.10)** | **1.65 (1.34, 2.04)** | **1.70 (1.38, 2.10)** | **1.63 (1.27, 2.09)** |
|  | When aged 40-50 years | **1.61 (1.32, 1.97)** | **1.60 (1.32, 1.96)** | **1.70 (1.40, 2.08)** | **1.63 (1.29, 2.09)** |
| Glucose | When aged 26-36 years | **1.46 (1.01, 2.11)** | 1.38 (0.99, 1.97) | **1.42 (1.00, 2.02)** | **1.49 (1.04, 2.26)** |
|  | When aged 40-50 years | **1.18 (1.01, 1.38)** | 1.17 (1.00, 1.37) | **1.18 (1.01, 1.38)** | 1.16 (0.97, 1.39) |
| Abbreviations: SBP (Systolic Blood Pressure), DBP (Diastolic Blood Pressure), BMI (body Mass Index), VMPA (total minutes of Vigorous or Moderate Physical Activity per week), TC (total cholesterol), HDL (High-Density Lipoprotein cholesterol), LDL (Low-Density Lipoprotein cholesterol)  *Number of fruit and vegetable serves per week  †Categorical variable of smoking (0 daily smoker, 1 weekly smoker, 2 monthly or less smoker, 3 non-smoker). Changes in smoking was calculated as the difference in these categories from baseline to follow-up. | | | | | |

| **Supplementary Table 4.** Logistic regression of risk score components with carotid plaque, with adjustment for changes over 13 years of follow-up | | | | | |
| --- | --- | --- | --- | --- | --- |
|  |  | Presence of carotid plaque | Number of carotid plaque  (None, one side or two sides) | Plaque thickness (mm)  (0; ≤1.5; 1.5-2; 2-2.5; >2.5) | Plaque area (mm^2^)  (0, ≤5, 5-10, 10-20, >20) |
| **Adjusting for changes over 13 years** | | OR (95% CI) | OR (95% CI) | OR (95% CI) | OR (95% CI) |
| SBP | SBP when aged 26-36 years | **1.03 (1.02, 1.05)** | **1.03 (1.02, 1.05)** | **1.03 (1.02, 1.05)** | **1.03 (1.01, 1.05)** |
|  | Changes in SBP over 13 years | 1.00 (0.99, 1.02) | 1.00 (0.99, 1.02) | 1.01 (0.99, 1.02) | 0.99 (0.97, 1.01) |
| DBP | DBP when aged 26-36 years | **1.04 (1.02, 1.06)** | **1.04 (1.02, 1.06)** | **1.04 (1.02, 1.06)** | **1.04 (1.01, 1.07)** |
|  | Changes in DBP over 13 years | **1.02 (1.01, 1.04)** | 1.02 (1.00, 1.04) | 1.02 (1.00, 1.04) | 1.01 (0.98, 1.03) |
| BMI | BMI when aged 26-36 years | **1.05 (1.01, 1.10)** | **1.05 (1.01, 1.09)** | **1.05 (1.01, 1.09)** | 1.02 (0.97, 1.08) |
|  | Changes in BMI over 13 years | 0.95 (0.88, 1.01) | 0.95 (0.89, 1.01) | 0.94 (0.89, 1.01) | 0.98 (0.85, 1.00) |
| Physical activity | VMPA when aged 26-36 years | 1.00 (1.00, 1.00) | 1.00 (1.00, 1.00) | 1.00 (1.00, 1.00) | 1.00 (1.00, 1.00) |
|  | Changes in VMPA over 13 years | 1.00 (1.00, 1.00) | 1.00 (1.00, 1.00) | 1.00 (1.00, 1.00) | 1.00 (1.00, 1.00) |
| Diet* | Diet when aged 26-36 years | 0.93 (0.82, 1.05) | 0.93 (0.82, 1.04) | 0.94 (0.84, 1.06) | 0.86 (0.74, 1.01) |
|  | Changes in diet over 13 years | 0.90 (0.80, 1.01) | 0.90 (0.81, 1.02) | 0.92 (0.83, 1.03) | 0.87 (0.75, 1.01) |
| Smoking† | Smoking when aged 26-36 years | 0.83 (0.64, 1.06) | 0.83 (0.65, 1.07) | 0.81 (0.64, 1.04) | **0.71 (0.54, 0.92)** |
|  | Changes in smoking over 13 years | 0.99 (0.74, 1.33) | 0.97 (0.73, 1.30) | 0.98 (0.74, 1.30) | 0.83 (0.60, 1.15) |
| TC | TC when aged 26-36 years | **1.58 (1.27, 1.97)** | **1.55 (1.25, 1.92)** | **1.65 (1.33, 2.05)** | **1.54 (1.18, 2.00)** |
|  | Changes in TC over 13 years | 1.20 (0.93, 1.56) | 1.21 (0.93, 1.55) | 1.29 (1.00, 1.64) | 1.20, 0.88, 1.64) |
| HDL | HDL when aged 26-36 years | **0.37 (0.19, 0.74)** | **0.39 (0.20, 0.76)** | **0.45 (0.24, 0.85)** | **0.26 (0.11, 0.64)** |
|  | Changes in HDL over 13 years | 0.61 (0.30, 1.26) | 0.61 (0.30, 1.22) | 0.65 (0.34, 1.27) | 0.58 (0.23, 1.44) |
| LDL | LDL when aged 26-36 years | **1.87 (1.45, 2.41)** | **1.84 (1.44, 2.36)** | **1.96 (1.53, 2.52)** | **1.88 (1.39, 2.54)** |
|  | Changes in LDL over 13 years | 1.15 (0.86, 1.54) | 1.18 (0.89, 2.36) | 1.27 (0.96, 1.68) | 1.23 (0.87, 1.75) |
| Glucose | Glucose when aged 26-36 years | **1.52 (1.04, 2.21)** | **1.42 (1.00, 2.05)** | **1.46 (1.01, 2.10)** | **1.55 (1.01, 2.40)** |
|  | Changes in glucose over 13 years | 1.07 (0.88, 1.30) | 1.05 (0.87, 1.29) | 1.05 (0.87, 1.28) | 1.03 (0.80, 1.32) |
| Abbreviations: SBP (Systolic Blood Pressure), DBP (Diastolic Blood Pressure), BMI (body Mass Index), VMPA (total minutes of Vigorous or Moderate Physical Activity per week), TC (total cholesterol), HDL (High-Density Lipoprotein cholesterol), LDL (Low-Density Lipoprotein cholesterol)  *Number of fruit and vegetable serves per week  †Categorical variable of smoking (0 daily smoker, 1 weekly smoker, 2 monthly or less smoker, 3 non-smoker). Changes in smoking was calculated as the difference in these categories from baseline to follow-up. | | | | | |

| **Supplementary Table 5.** Net Reclassification of adding PCE to FBS in prediction of carotid plaque at follow-up  (NRI estimate −0.0062, p=0.88) | | | | | | |
| --- | --- | --- | --- | --- | --- | --- |
|  |  |  | Risks of carotid plaque estimated by FBS + PCE | | | |
|  |  |  | <10% | 10-15% | ≥15% | Total |
| Risks of carotid plaque estimated by FBS at baseline | Presence of plaque at follow-up | <10% | 17 | 1 | 0 | 18 |
|  |  | 10-15% | 0 | 43 | 2 | 45 |
|  |  | ≥15% | 0 | 16 | 32 | 48 |
|  |  | Total | 17 | 60 | 34 | 111 |
|  | Absence of plaque at follow-up | <10% | 202 | 10 | 0 | 212 |
|  |  | 10-15% | 23 | 267 | 19 | 309 |
|  |  | ≥15% | 0 | 86 | 114 | 200 |
|  |  | Total | 225 | 363 | 133 | 721 |
| Abbreviations: FBS (Fuster-BEWAT Score), PCE (Pooled Cohort Equations) | | | | | | |

| 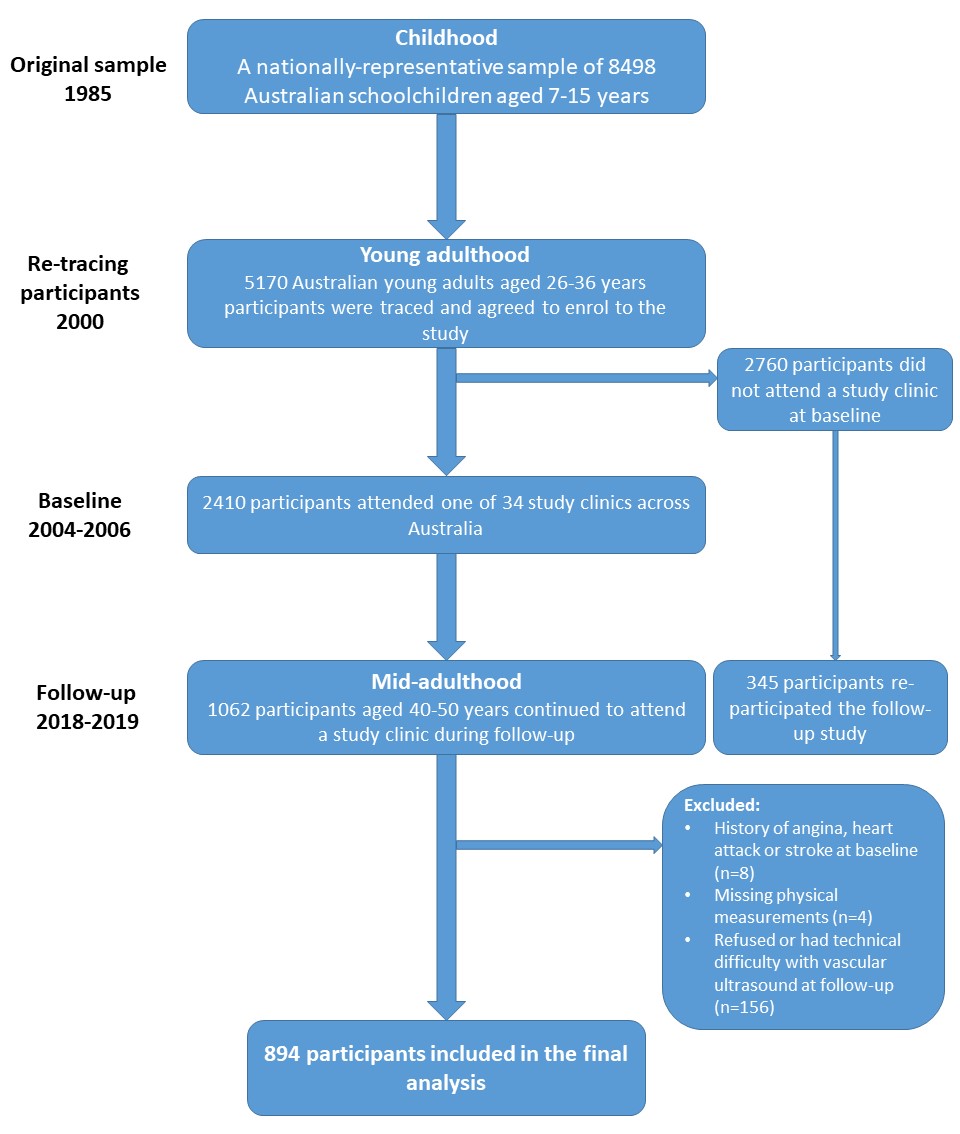 |
| --- |
| **Supplementary Figure 1.** Study sample |

| 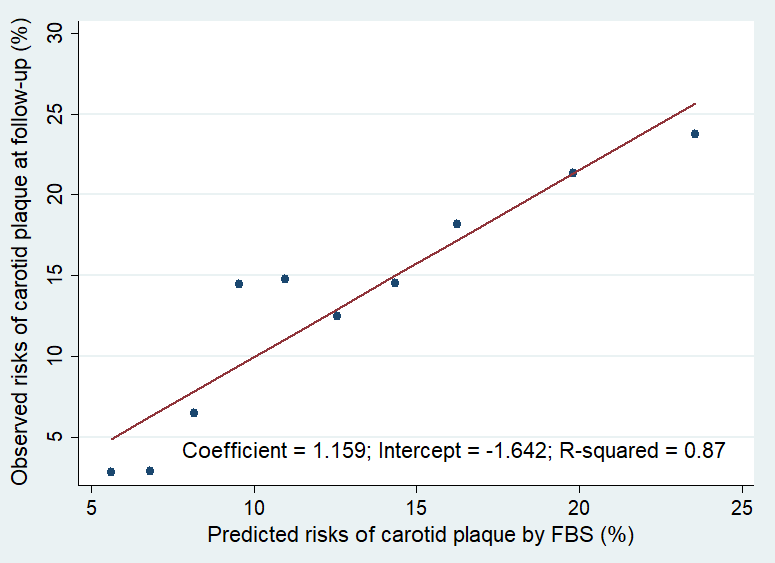 |
| --- |
| 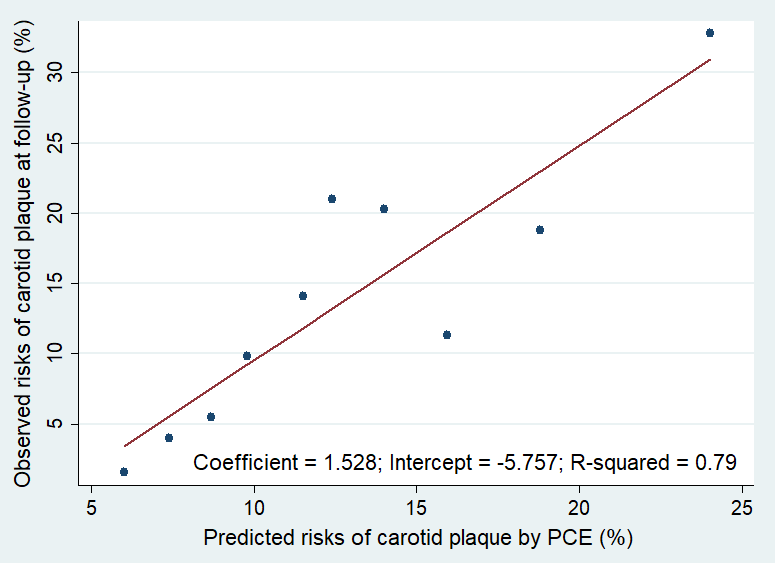 |
| **Supplementary Figure 2.** Calibration of the 3 risk scores in predicting carotid plaque at follow-up |
